# Extracellular Vesicle MicroRNA Transfer in Lung Diseases

**DOI:** 10.3389/fphys.2017.01028

**Published:** 2017-12-12

**Authors:** Jie Chen, Chengping Hu, Pinhua Pan

**Affiliations:** Department of Respiratory and Critical Care Medicine, Key Site of National Clinical Research Center for Respiratory Disease, Xiangya Hospital, Central South University, Changsha, China

**Keywords:** extracellular vesicle, exosome, microvesicle, microRNA, lung disease

## Abstract

MicroRNAs (miRNAs) are single-stranded, small non-coding RNAs that ate involved in the transcriptional and post-transcriptional regulation of gene expression. Recently, miRNAs were demonstrated to be effectively delivered to a target cell or tissue from a host cell via extracellular vesicles (EVs). These EVs can be detected in blood, urine, exhaled breath condensates, bronchoalveolar lavage fluid (BALF), and other fluids. miRNAs are generated by donor cells and then packaged into EVs and delivered with intact functionality. After being delivered to the target cells, they regulate the translation of their target genes and the function of the target cells. Thus, EV transported miRNAs have become a new method for intercellular communication. EV miRNA transfer is well-documented in various pulmonary diseases, such as chronic obstructive pulmonary disease (COPD), asthma, pulmonary hypertension, and acute lung injury (ALI). In this review, we summarize the novel findings of EV miRNA transfer, focusing on the roles of miR-210, miR-200, miR-17, miR-146a, miR-155, and other miRNAs that are transported from primary human bronchial epithelial cells (HBECs), BALF, mesenchymal stem cells, and dendritic cells.

## Introduction

Extracellular vesicles (EVs) consist of a small lipid bilayer surrounding vesicles containing proteins, lipids, metabolites, and nucleic acids (Yáñez-Mó et al., 2015). EVs are released by many cell types, including bacteria and plant cells, which makes cross-kingdom communication possible (Colombo et al., 2014). They can be detected in blood, urine, saliva, exhaled breath condensates, bronchoalveolar lavage fluid (BALF), and other fluids (Colombo et al., 2014). The release of EVs was initially considered to be a process of discarding membrane proteins in cells (Pan and Johnstone, 1983). However, the following studies have demonstrated that the release of EVs is a critical mediator of cell-to-cell communication, which is involved in the physiological and pathological processes of different diseases.

MicroRNAs (miRNAs), small non-coding RNAs, are involved in the transcriptional and post-transcriptional regulation of protein-coding gene expression (Bartel, 2004). In the past few years, an increasing number of studies have reported the role of miRNAs in the development of lung diseases (Alipoor et al., 2016). However, many of them have concentrated on the effects of miRNA' regulation in only the primary cells where the miRNA was produced (Martinez-Nunez et al., 2011; Ebrahimi and Sadroddiny, 2015).

More recently, many studies have begun to report the effects of miRNA transfer via EVs (Das and Halushka, 2015; Claßen et al., 2017). In respiratory diseases, this transfer was shown to be mediated via intercellular communication between many types of cells in the respiratory system: endothelial cells (ECs), (Aliotta et al., 2016; Serban et al., 2016) bronchial epithelial cells (BECs), (Fujita et al., 2015) dendritic cells (DCs), (Alexander et al., 2015) mesenchymal stem cells (MSC),(Lee et al., 2012) and others (Table 1). Since EVs contain almost the same cell surface proteins with their origin cells, they can fuse to the target recipient cells (Raposo and Stoorvogel, 2013). When those EVs were absorbed by recipient cells, they will transfer a variety of biological molecules including miRNAs. This process especially EV miRNA transfer will alter the biological activities of recepient cells and influence microenvironment of the lungs (Huang-Doran et al., 2017; Nana-Sinkam et al., 2017). Here, we aim to summarize the novel findings of EV miRNA transfer in different lung diseases and explore their potential clinical significance.

**Table 1:** Known EV transfer of miRNAs between lung cells.

**miRNA(s)**	**Source**	**Donor cell**	**Recipient cell**	**Target gene**	**Reference**
miRNA-210	Cell culture	BEC	Fibroblast	ATG7	Fujita et al., 2015
miRNA-191,miRNA-126, miR125a	Cell culture	EC	Macrophages		Serban et al., 2016
miR-204	Mouse lung	MSC	Lung immune cells and immune cells	STAT3 pathway	Lee et al., 2012
miR-146a, miR-155	Cell culture	DC	Immune cell		Alexander et al., 2015

*BEC, bronchial epithelial cell; EC, endothelial cell; MSC, mesenchymal stem cell; DC, dendritic cell*.

## EV biogenesis

EVs are generally known to mediate normal physiological homeostasis and pathological processes by facilitating intercellular communication (Huang-Doran et al., 2017; Nana-Sinkam et al., 2017). As the research progresses, there appear to be many definitions for EVs. According to their origin, EVs are usually categorized into exosomes and microvesicles (Colombo et al., 2014).

Exosomes are round vesicles 30–100 nm in diameter that originate from intracellular endosomes and subsequently form multivesicular bodies (MVBs) (Raposo and Stoorvogel, 2013; Colombo et al., 2014). Exosomes contain a collection of endosome-associated proteins (including Rab GTPase, SNAREs, Annexins, and flotillin), some of which are contributed to MVBs biogenesis (TSG101 and annexins). Besides, transmembrane or lipid-bound extracellular proteins (CD9, CD63, CD81, cell adhesion molecules, and growth factor receptors), cholesterol, sphingomyelin, and hexosylceramides are also demonstrated to be enriched on exosomes (Raposo and Stoorvogel, 2013; Lötvall et al., 2014). In addition to proteins, cholesterol and lipids, exosomes also contain a large number of DNA, mRNA, miRNA, and other small non-coding RNA. The release of exosomes was demonstrated to be controlled by RAB11, RAB27A/B, and RAB35 (Hsu et al., 2010; Ostrowski et al., 2010).

In contrast, microvesicles, also named ectosomes or membrane particles or microparticles, are small cytoplasmic protrusions that directly bud off of the plasma membrane (Raposo and Stoorvogel, 2013; Chen et al., 2015). They have an irregular morphology and a wide size, with a diameter of 100–1,000 nm (Raposo and Stoorvogel, 2013). Due to their different modes of formation, microvesicles are usually larger than exosomes and do not lipid-bound extracellular proteins or cytosolic proteins or cholesterol (Raposo and Stoorvogel, 2013). However, in similar with exosomes, microvesicles also contain a large number of DNA, mRNA, miRNA, and other small non-coding RNA. Microvesicle release was shown to be related to the ADP-ribosylation factor 6 (ARF6) GTP/GDP cycle (Muralidharan-Chari et al., 2009).

Despite their different origin, composition and release, exosomes and microvesicles share a similar density and a somewhat similar size, which makes it difficult to separate them using ultracentrifugation or purely on the basis of their size (Das and Halushka, 2015).

## miRNA biogenesis

MiRNAs are small, non-coding RNAs with a sequence length between 18 and 25 nucleotides (nts), and they serve as master regulators of translation (Bartel, 2004). The levels of miRNA expression change according to cell stressors, and they are likely important in the progression and maintenance of lung diseases (Sessa and Hata, 2013).

**Figure 1:**
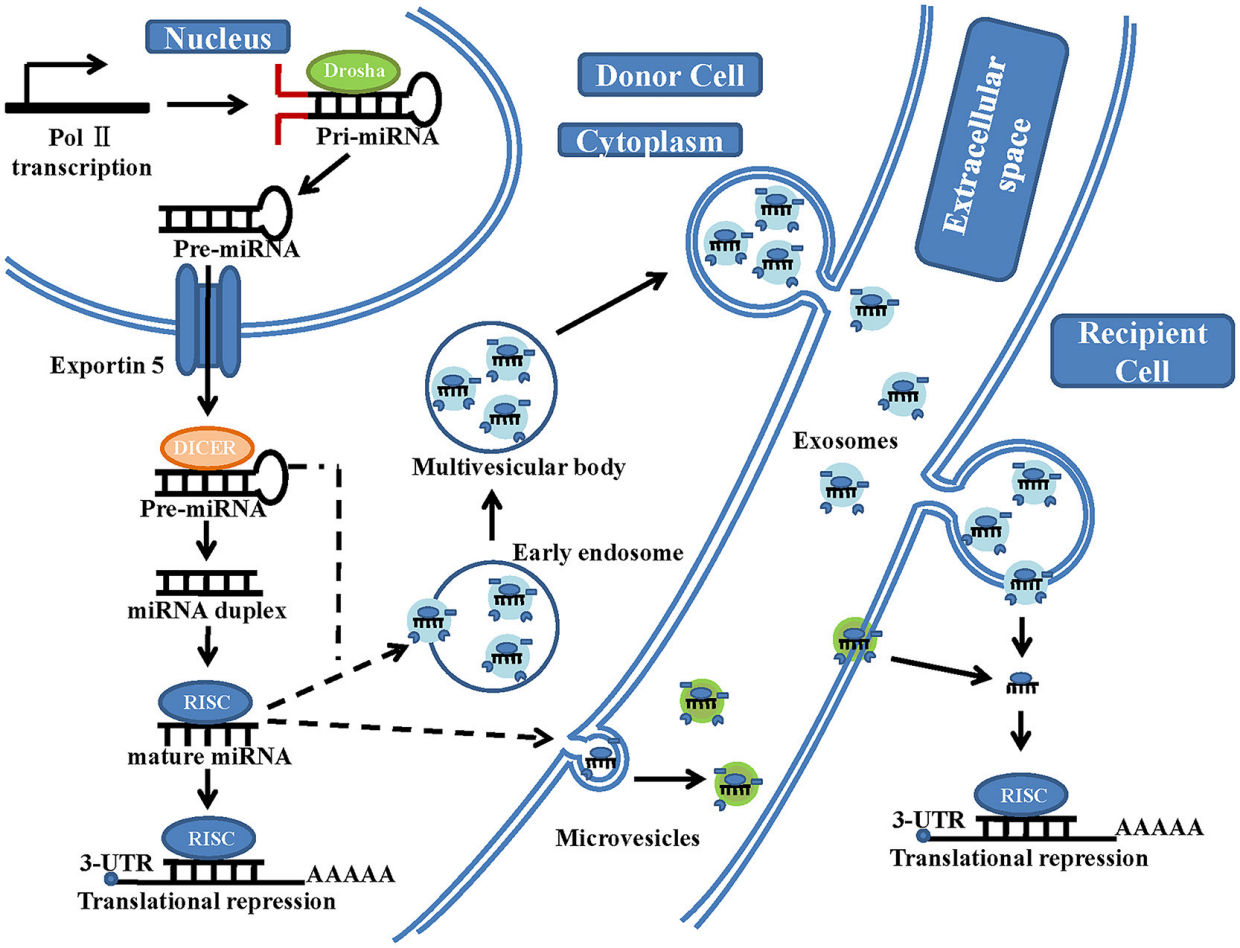
A schematic illustration of microRNA biogenesis and extracellular vesicle microRNA transfer. In the nucleus of the donor cells, miRNA genes are transcribed by RNA polymerase II (Pol II), forming a primary transcript (pri-miRNA). This is then processed by the RNase III-type endonuclease family protein Drosha into a precursor molecule (pre-miRNA). It is then exported to the cytoplasm by the transport protein exportin-5. In the cytoplasm, pre-miRNA is further processed by another RNase III-type endonuclease family protein, Dicer, which generates an intermediary miRNA duplex, of which one strand is loaded onto the RNA-induced silencing complex (RISC) to form mature miRNA. On the one hand, these mature miRNA can be directly transferred to the recipient cells by microvesicles, which are shed from the plasma membrane. On the other hand, these mature miRNA and some pre-miRNA can be loaded into the multivesicular bodies (MVBs), which are generated via early-endosomal membrane invagination. These MVBs then dock onto the cell membrane and release positive exosomes into the extracellular space (including serum and other biological fluids). The exosomal fusion with the plasma membrane of the recipient cell, or phagocytosis followed by membrane fusion, leads to the release of miRNA cargo into the cytosol and translational repression.

The generation of mature miRNAs is a complicated process (Figure 1). Within the nucleus, miRNA genes are first transcribed by RNA polymerase II to generate long primary transcripts (pri-miRNAs) (Sessa and Hata, 2013). These pri-miRNAs are subsequently cleaved by Drosha (RNase III enzyme) into hairpin-like structures called pre-miRNAs (Sessa and Hata, 2013; Lui et al., 2015). The pre-miRNAs are terminally transported to the cytoplasm by exportin-5, further cleaved by Dicer (another RNase III-type endonuclease) in complex with accessory dsRNA-binding proteins and formed into a mature dsRNA duplex (Gwizdek et al., 2003). Then, the two strands of the mature duplex are separated. One of them (called the passenger strand) is degraded, while the other one (called the functional or guide strand) is preferentially loaded onto the Argonaut family protein Ago2, which is incorporated in the RNA-induced silencing complex (RISC) (Bartel, 2004). The guide strand of miRNA directs Ago2 proteins to the 3'untranslated regions (UTRs) of the target mRNA with their 6-nt seed-match site (Bartel, 2004). When the binding between miRNAs and the mRNA target sequences is partially complementary, translation will be suppressed, and the protein levels of the target gene will be reduced. In contrast, when the binding is perfectly complementary, the target mRNA strand will be degraded (Bartel, 2004). However, Lim et al. showed that partially complementary binding (between miRNAs and their mRNA target sequences) can also cause mRNA degradation (Lim et al., 2005). Only 7 nt sequences of miRNA are needed to bind to their target mRNA; therefore, a single miRNA can regulate numerous different genes, (Bartel, 2004) and studies on the regulatory functions of each miRNA are ongoing (Bartel, 2004).

Recently, in addition to exploring the biogenesis of miRNA, many studies have tried to create an itemized list of all the miRNAs and identify their localization. To assign stable and consistent names to newly discovered miRNAs, the primary miRNA sequence repository database, miRBase was established (Kozomara and Griffiths-Jones, 2014). Currently, the latest miRBase release contains nearly 2,800 human miRNAs; however, according to recent studies, there are still many miRNAs to be discovered (Londin et al., 2015). Although there are many miRNAs in cells, only a few of them can reach high enough levels to regulate the synthesis of proteins. (Mullokandov et al., 2012) It was demonstrated that different cell types, and even the same cell type in different cell states, expressed different miRNAs (Mullokandov et al., 2012). To prevent confusion, a specific naming system for miRNAs has been established. The naming system includes the species, the miR and the hairpin (“−5p” or “3p”).

## miRNAS in EVs are transferred between cells

miRNAs, recognized as helpful biomarkers of diseases, have been described as being active EV cargo (Tkach and Théry, 2016). They are generated by donor cells and then packaged into EVs and delivered with intact functionality. After being delivered to the target cells, the miRNAs regulate the translation of the target genes and the function of the target cells (Figure 1) (Tkach and Théry, 2016). This idea was first described by Valadi et al. (2007). They found that exosomes derived from either human or mice mast cells contained many specific mRNAs and miRNAs, and these could be transported to other mast cells (Valadi et al., 2007). Following this study, hundreds of other studies have reported similar findings. Ong et al. reported that exosomes purified from cardiac endothelial cells overexpressing HIF-1 delivered miR-126 and miR-210 to recipient cardiac progenitor cells (CPCs). They reported that those microRNAs induced a glycolytic switch in the CPCs, which would be helpful for tolerating a hypoxic stress state (Ong et al., 2014). Ding et al. reported that exosomes derived from pancreatic cancer cells could deliver miR-212-3p to dendritic cells and inhibit RFXAP expression (Ding et al., 2015). Viñas et al. revealed that exosomes derived from human cord blood endothelial colony forming cells delivered miR-486-5p to endothelial cells, targeting the PTEN/Akt pathway. Their results suggested that the use of human miR-486-5p containing exosomes could be a viable strategy for protecting against endothelial injury in ischemic kidney injury (Viñas et al., 2016).

Since an increasing number of specific miRNAs have been found in EVs, their selective mechanisms have attracted great attention. It was reported that one of the mechanisms was related to a ribonucleoprotein (hnRNPA2B1) combined with GGAG motifs on miRNAs. This mechanism could determine which miRNAs are selected for incorporation into exosomes (Villarroya-Beltri et al., 2013). In addition, it was also demonstrated that knockout of AGO2 (a gene related to the RISC complex) could inhibit specific miRNAs in EVs (Guduric-Fuchs et al., 2012). In addition to EVs, there is other evidence support the notion that miRNA cellular transfer can also occur via high-density lipoproteins (Rotllan et al., 2016). However, due to the complexity of mechanisms of the miRNA transfer, this possibility remains to be verified.

## EV miRNA transfer in lung diseases

In the past few years, our understanding of the function of miRNA in lung diseases has grown gradually. The role that miRNAs play in their original cells is thought to be important in lung biology and disease pathogenesis (Sessa and Hata, 2013). Recently, increasing attention has been forced on the role of miRNA regulation of recipient cells via EV transfer (Pattarayan et al., 2016). Although a great number of miRNAs have been demonstrated to be included in EVs, it was not completely the same as that in EV origin cells. Some specific miRNAs are selectively exported to EVs, while others are excluded (Nolte-'t Hoen et al., 2012). This sorting signals and mechanisms seem to be complex. Gibbings et al. reported that GW182, a component of RISC, may contribute to the loading of miRNAs into multivesicular bodies (Gibbings et al., 2009). Then Villarroya-Beltri et al. demonstrated that miRNA sorting into exosomes was related with sumoylated heterogeneous nuclear ribonucleoprotein A2B1 (Villarroya-Beltri et al., 2013). In addition, Hagiwara et al. also demonstrated that miRNA sorting into EVs was mediated by Annexin A2 (Hagiwara et al., 2015).

A great number of studies show that the number and contents of EVs change according to different diseases and disease status (Raposo and Stoorvogel, 2013; Colombo et al., 2014). Based on the available evidence, miRNAs are diferentially enriched in EVs, and their expression patterns also change with respect to different lung diseases (Pattarayan et al., 2016). Therefore, EV miRNAs are considered to be potential diagnostic biomarkers of pulmonary diseases (Pattarayan et al., 2016). In addition, EV miRNAs can be delivered to recipient cells (including respiratory cells and immunological cells) and then alter their status and biological processes, which will affect the pathophysiology of lung diseases (Kubo, 2017; Nana-Sinkam et al., 2017).

## EV miRNA transfer in COPD

Chronic obstructive pulmonary disease (COPD) is an inflammatory respiratory disease that is induced by chronic exposure of the airway to irritants, including cigarette smoke and other noxious particles (Vestbo et al., 2013). The pathology of COPD is characterized by epithelial cell injury, damage to the pulmonary capillary vasculature, acceleration of epithelial cell senescence, and airway remodeling (Vestbo et al., 2013). An increasing number of studies have demonstrated that injured cells, such as epithelial and endothelial cells, contribute greatly to the pathophysiology of COPD,(Takahashi and Kubo, 2014) because they can secrete chemical agents that take part in modulating systemic immune responses (Takahashi and Kubo, 2014).

Endothelial cell injury within the lung parenchyma is an important factor in emphysema (Kratzer et al., 2013). The injured endothelial cells release different types of EVs, which are involved in coagulation, inflammation, endothelial function, and angiogenesis (Kadota et al., 2016). Gorden et al. showed that the number of circulating endothelial cell-derived microparticles in smokers with early emphysema was significantly increased (Gordon et al., 2011). Takahashi et al. also demonstrated that some types of endothelial cell-derived microparticles in COPD patients (especially in patients with exacerbation COPD) were significantly increased compared to those in healthy volunteers (Takahashi et al., 2012). In addition, bronchial epithelial cells (BECs), which are located in the airway lumen, play an essential role in protecting the airway epithelium structural and functional damage (Kadota et al., 2016). When injured by environmental stimuli such as cigarette smoke, BECs release proinflammatory factors, such as TNF-α, IL-1β, GM-CSF, TGFβ, and CXCL-8, which may act in both an autocrine and paracrine manner (Kadota et al., 2016). They are also recognized to be the main source of exosomes in the lung (Kulshreshtha et al., 2013).

Furthermore, conclusive evidence of EV-mediated miRNA transfer and miRNA functioning as a modulator of intercellular crosstalk and COPD pathogenesis was reported by Fujita et al. They observed that the levels of miR-210 expression in both BECs and BEC-derived EVs were significantly increased after CSE exposure. Moreover, they demonstrated that miR-210 in BEC-derived EVs could be transferred into lung fibroblasts and inhibited ATG7 expression and induced myofibroblast differentiation (Fujita et al., 2015). The research indicated that CSE exposure changed the composition of BEC-derived EVs and recognized miR-210 as an autophagy factor in myofibroblast differentiation (Fujita et al., 2015).

Another study exploring EV transfer of miRNAs in the pathogenesis of COPD was done by Serban et al. (2016). They found that the secretion of microparticles depended on the ceramide-synthesis enzyme acid sphingomyelinase (aSMase). They observed that the activity of aSMase in both COPD patients and a COPD mouse model was obviously increased. They also demonstrated that cigarette smoke induced the endothelial cell release of microparticles with special miRNAs, such as miR-191, miR-126 and miR125a, via aSMase. Those miRNAs were transferred to macrophages and promoted the clearance of apoptotic cells (Serban et al., 2016).

## EV miRNA transfer in asthma

Asthma is a chronic inflammatory pulmonary disorder with reversible airway narrowing and airway hyperresponsiveness induced by many types of stimuli, such as air pollutants and allergens (Lambrecht and Hammad, 2015). The main pathophysiological characteristics of asthma are due to proinflammatory factors (for example, IL-4, IL-5, and IL-13) that are released by many immune cells (Lambrecht and Hammad, 2015). Expression of these factors results in increased numbers or increased activation of mast cells and increased eosinophils, along with airway remodeling, reversible airway hyperresponsiveness, and airway obstruction (Fujita et al., 2014).

EVs are secreted by many cells that may be related to asthma, including lymphocytes, bronchial epithelial cells, and immune cells. In 2003, Skokos et al. found that mast cell-derived exosomes promoted the maturation of DC and helped their Ag-presenting function (Skokos et al., 2003). Kulshreshtha et al. also reported that EVs derived from BECs played a critical role in the pathogenesis of asthma. They found that IL-13 (a critical cytokine in asthma) could change the composition and quantity of BEC-derived EVs. During allergic inflammation, the production of those EVs would be increased. Importantly, Kulshreshtha et al. demonstrated that exosomes derived from IL-13-induced BECs could stimulate macrophage proliferation and worsen asthmatic conditions (Kulshreshtha et al., 2013). Recently, Mazzeo et al. found that exosomes derived from eosinophils could also contribute to the development of asthma. They demonstrated that eosinophil-derived exosomes were increased in asthmatic patients and could modulate the features of asthma *in vitro* after transferred to recipient cells (Mazzeo et al., 2015). In addition, several reports indicated that EVs derived from BALF played a critical role in modulating immune responses during asthma (Admyre et al., 2003; Prado et al., 2008). Admyre et al. first purified exosomes from BALF and demonstrated that those exosomes carried co-stimulatory molecules. They suggested that the exosomes could deliver antigens to the immune system and modulate immune reactions (Admyre et al., 2003).

In the field of immunology, the earliest studies of EV miRNA transfer that are related to asthma are from Mittelbrunn et al. (2011). They demonstrated that there were distinct miRNAs in the exosomes from immune cells and that some miRNAs could be transferred into the antigen-presenting cells. Then, in 2012, Montecalvo et al. found that exosomes that were released from DCs delivered their content (like miRNAs) to the DC cytosol (Montecalvo et al., 2012). Moreover, Levänen et al. observed that miRNAs in BALF exosomes were different between asthmatic patients and controls (Levänen et al., 2013). Those miRNAs were evaluated by microarrays, which showed that 24 miRNAs were significantly altered between those two groups (Levänen et al., 2013). Furthermore, they also demonstrated that the expression profiles of those 24 miRNAs were highly correlated with forced expiratory volume in 1 s (FEV1) in asthmatic patients.

## EV miRNA transfer in PAH

Pulmonary arterial hypertension (PAH) consists of a group of debilitating, incurable diseases characterized by progressive vascular narrowing and elevations in the mean pulmonary artery pressure. It ultimately leads to right-sided heart failure and death (Rich et al., 1987). The pathological changes of PAH include pulmonary artery endothelial cell (PAEC) proliferation; pulmonary artery smooth muscle cell (PASMC) proliferation, migration, and contraction; inflammation; and fibroblast proliferation, activation, and migration (Zhou et al., 2015). Although advancements in the pathogenesis and treatment of PAH have been made, it remains a severe and devastating disease with a poor prognosis and a high mortality (Calway and Kim, 2015).

The role of EVs derived from PAH patients is well-established. Several groups reported increased numbers of circulating EVs in PAH patients compared to healthy subjects (Amabile et al., 2013; Belik et al., 2016). Interestingly, Belik et al. found that the levels of endoglin-positive endothelium-derived microparticles in patients with chronic thromboembolic pulmonary hypertension (CTEPH) were higher than those in healthy subjects. They showed that these microparticles contributed to promoting survival and angiogenesis in PAEC (Belik et al., 2016). In addition, Bakouboula et al. showed the number of endothelium-derived microparticles containing CD105 in PAH patients was much greater than that in the controls. They further demonstrated that there were more procoagulant microparticles in the blood from the pulmonary artery than in that from the jugular vein of PHA patients (Bakouboula et al., 2008).

The earliest study of EV miRNA transfer in PAH was reported by Lee et al. (2012). They demonstrated that mesenchymal stem cell (MSC) exosomes blocked right ventricular hypertrophy. More interestingly, they found that MSC exosomes suppressed the hypoxic activation of STAT3 and un-regulated the levels of miR-204, which could be inhibited by STAT3. These findings open exciting avenues given their relevance to the following studies. In a recent investigation, Aliotta et al. demonstrated that exosomes that were released by pulmonary vascular endothelial cells in PAH contained increased levels of miRNAs that are known to promote PAH. However, treatment with MSC exosomes blocked the development of PAH, because they contained high levels of anti-inflammatory, anti-proliferative miRNAs (Aliotta et al., 2016). More research should be conducted to explore the specific mechanism of EV miRNA in PAH.

## EV miRNA transfer in ALI

Acute lung injury (ALI) and acute respiratory distress syndrome (ARDS) develop in response to sepsis, trauma, pneumonia, and gastric content aspiration (Matthay and Zemans, 2011). ALI and ARDS are characterized by widespread uncontrolled inflammation in the lungs that is associated with the loss of surfactant and impaired pulmonary capillary endothelial barriers, leading to fluid accumulation in the distal airspaces (Simonson et al., 2015). Although great progress has been made regarding the treatments for ARDS, ARDS mortality is still high (34.9% for mild, 40.3% for moderate and 46.1% for severe ARDS) (Bellani et al., 2016).

To date, a few groups have studied the therapeutic effects of EVs in ALI (Zhu et al., 2014; Monsel et al., 2015; Moon et al., 2015). Zhu et al. demonstrated a biologic effect of MSC microvesicles in endotoxin-induced ALI mice (Zhu et al., 2014). MSC microvesicles have almost the same protective effects as MSCs themselves in repairing injured lung tissues. KGF mRNA knockdown partially abrogated the protective effects of MSC microvesicles, indicating that the KGF protein plays a central role in the process of lung repair. Monsel et al. (2015) demonstrated that the administration of MSC microvesicles increased the survival rate and inhibited lung inflammation and bacterial growth in animals with *Escherichia coli* pneumonia. In addition, some groups have also explored the functions of EVs in the pathogenesis of ALI (Letsiou et al., 2015; Moon et al., 2015). Moon et al. (2015) demonstrated that hyperoxia stimulated the formation/release of lung epithelial cell EVs. Those EVs activated macrophages and subsequently promoted neutrophil infiltration and lung inflammation in ALI. Letsiou et al. reported that endothelial micropaticles represent a novel diagnostic, prognostic, and therapeutic tool that is potentially linked to disease pathogenesis, severity, and outcome in ALI (Letsiou et al., 2015).

Since miRNAs have been reported to be involved in the pathophysiology of ALI, the transfer of EV miRNAs to many types of lung cells may be an interesting, novel pathomechanism (Cai et al., 2012). The best-studied miRNA that participates in the mechanism of ALI is miR-146a, (Zeng et al., 2013) which can repress the expression of IRAK-1 and TRAF-6, and suppress inflammatory mediators. Recently, Alexander et al. (2015) observed that exosomes that were derived from dendritic cells contained miR-146a and miR-155, which could be transferred between immune cells *in vivo*. They also demonstrated that exosomes with miR-146a reduced the expression of inflammatory genes, while miR-155 promoted this process. EV miRNA transfer in ALI clearly deserves further exploration, which will provide additional insight into the underlying mechanism of ALI.

## EV miRNA transfer in other lung diseases

In addition to the abovementioned lung diseases, the role of EV miRNA transfer in some other lung disorders has also been investigated. There are few therapeutic options for idiopathic pulmonary fibrosis (IPF), and it has a dismal median survival of 2–3 years (Ley et al., 2011). Recently, Makiguchi et al. reported that the expression of serum EV miR-21-5p was significantly increased in both IPF mouse models and patients (Makiguchi et al., 2016). Moreover, they revealed that the expression of miR-21-5p in serum EVs was related to IPF progression and mortality (Makiguchi et al., 2016).

Pulmonary tuberculosis (TB) is a serious infectious disease that causes heavy economic burden in undeveloped countries (Mirsaeidi et al., 2015). Membrane vesicles derived from *Mycobacterium tuberculosis* (MTB) and other mycobacteria contain immune modulating molecules that are beneficial to the bacterium (Prados-Rosales et al., 2011). Comprehensive proteomic-based analysis has been used to determine the protein content of exosomes derived from macrophages infected with either live or dead MTB *in vitro* (Giri et al., 2010). This analysis revealed the dominant presence of host proteins along with 41 mycobacterial proteins within the secreted exosomes (Giri et al., 2010). Subsequent studies identified 249 new proteins via several LC-MS/MS analyses of MTB EVs (Lee et al., 2015). Recently, Singh et al. reported that there were 57 unique miRNAs in exosomes that were secreted by MTB-infected macrophages (Singh et al., 2015). These data support the functional and diagnostic potential of EV miRNAs in tuberculosis.

Obstructive sleep apnea (OSA) is a pulmonary disorder caused by complete or partial obstructions of the upper airway. It leads to episodes of intermittent hypoxia (Punjabi et al., 2009). Several reports have demonstrated that the amounts of EVs (such as endothelium micropaticles and platelet micropaticles) are increased in patients with OSA (Kim et al., 2011; Maruyama et al., 2012). Trzepizur et al. reported that circulating micropaticles in OSA patients induce endothelial dysfunction (ED) *in vivo* (Trzepizur et al., 2014). Recently, Khalyfa et al. isolated plasma-derived EVs from pediatric patients with OSA or OB without OSA and with the presence or absence of ED. They found that the expression of miRNA-630 in EVs was decreased in children with ED (Khalyfa and Kheirandish-Gozal, 2016). In addition, they further studied plasma exosomes miRNA and endothelial function in healthy adults with intermittent hypoxia (Khalyfa et al., 2016). It was shown that intermittent hypoxia altered EV cargo in the circulation and that many circulating EV miRNAs contributed to cardiovascular dysfunction associated with OSA.

## Clinical trials and future directions

EV miRNA transfer studies are interesting since they may unveil new avenues and therapeutic targets for treating diseases. Their clinical effects have been explored in phase I and II trials diseases. For example, it was reported that tumor antigen-loaded DC-derived exosomes were efficacious and safe for treating non-small cell lung cancer (NSCLC) patients with boosting antitumor immunity (Besse et al., 2016). Additionally, Kordelas et al. showed that MSC-derived exosomes therapy could relieve the symptoms of steroid-refractory graft-vs.-host disease patients without any noticeable side effects (Kordelas et al., 2014).

However, the following issues should be focused on in the future before the extensive implementation of EV miRNAs in clinics. First, an improved classification system for EVs will be required. In addition, a defined, standardized protocol used to isolate EVs will also be required. The efforts made by the International Society of Extracellular Vesicles (ISEV) will promote the standardization of isolation methods and identification of EVs (Witwer et al., 2013). EV specific miRNAs also need to be extracted using routine methods that are different from those used to extract cellular miRNAs so that their levels can be used to assess the function of EVs. Second, the mechanisms that regulate incorporation of particular miRNA into EVs are still unknown. Although recent reports have presented evidence for incorporating particular miRNAs into EVs, (Cheng et al., 2014; Iftikhar and Carney, 2016) efforts should be made to identify the conditions that cause unique miRNAs to be packaged into EVs. Third, the mechanisms for recipient cell uptake of EV miRNAs remain unclear. More attention should be paid to EV miRNAs uptake mechanisms and the membrane receptors of those recipient cells (Iftikhar and Carney, 2016).

## Conclusion

The reports described above reflect only the first wave of discovery in this fascinating field. EVs could be derived from almost all respiratory cell types, including structural and immune cells, and they have been demonstrated to be important players in several cellular processes. EVs have been recognized as a tool for packaging miRNAs and delivering them with intact functionality. A great number of studies should be conducted regarding EV miRNA transfer in lung diseases. Based on the present evidence, EV miRNAs provide novel diagnostic biomarkers for a broad range of pulmonary diseases and may also be used for therapeutic interventions.

## Author contributions

JC: drafted, prepared, and edited the manuscript. CH: reviewed and edited the manuscript. PP: revised and edited the manuscript.

### Conflict of interest statement

The authors declare that the research was conducted in the absence of any commercial or financial relationships that could be construed as a potential conflict of interest.
